# Day 100 Absolute Monocyte/Lymphocyte Prognostic Score and Survival Post Autologous Peripheral Blood Hematopoietic Stem Cell Transplantation in Diffuse Large B-Cell Lymphoma

**DOI:** 10.4236/jct.2013.48153

**Published:** 2013-10

**Authors:** Ana I. Velazquez, David J. Inwards, Stephen M. Ansell, Ivana N. Micallef, Patrick B. Johnston, William J. Hogan, Svetomir N. Markovic, Luis F. Porrata

**Affiliations:** Division of Hematology, Department of Internal Medicine, Mayo Clinic, Rochester, USA

**Keywords:** Monocyte/Lymphocyte Prognostic Score, Diffuse Large B-Cell Lymphoma, Survival, Autologous Peripheral Blood Hematopoietic Stem Cell Transplantation

## Abstract

Day 100 prognostic factors post-autologous peripheral blood hematopoietic stem cell transplantation (APBHSCT) to predict clinical outcomes in diffuse large B-cell lymphoma (DLBCL) patients have not been studied. Thus, we retrospectively examined if day 100 absolute monocyte/lymphocyte prognostic score (AMLPS-100) affects clinical outcomes by landmark analysis from day 100 post-APBHSCT in DLBCL. Only DLBCL patients in complete remission at day 100 post-APBHSCT were evaluated. From 2000 to 2007, 134 consecutive DLBCL patients are qualified for the study. Patients with a day 100 absolute monocyte count (AMC-100) ≥ 630 cells/μL and day 100 absolute lymphocyte count (ALC-100) ≤ 1000 cells/μL experienced inferior overall survival (OS) and progression free survival (PFS). On multivariate analysis, the AMC-100 and ALC-100 remained independent predictors of OS and PFS. Combining both values into the AMLPS-100, the 5-year OS rates for low, intermediate, and high AMLPS-100 risk groups were 94% (95% CI, 83.0% – 98.1%), 70% (95% CI, 58.6% – 80.1%), and 13% (95% CI, 3.4% – 40.5%), respectively; and the 5-year PFS rates were 87% (95% CI, 74.0% – 94.1%), 68% (95% CI, 56.0% – 77.8%), and 13% (95% CI, 3.4% – 40.5%), respectively. The AMLPS-100 is a simple biomarker score that can stratify clinical outcomes from day 100 post-APBHSCT in DLBCL patients.

## 1. Introduction

Day 100 visit after stem cell transplantation is the current standard first follow-up visit to assess treatment response. Day 100 absolute lymphocyte count (ALC-100) [1], day 100 absolute monocyte count (AMC-100) [1], day 100 platelet count [2], graft versus host disease [3], and day 100 full donor chimerism [4] are day 100 prognostic factors related to clinical outcomes in allogeneic stem cell transplantation. In autologous peripheral blood hematopoietic stem cell transplantation (APBHSCT), multiple myeloma documented minimal residual disease at day 100 was associated with inferior survival. Nevertheless, prognostic factors to assess prognosis for diffuse large B-cell lymphoma (DLBCL) patients achieving a complete remission at day 100 post-APBHSCT have not been evaluated. We previously reported that the absolute lymphocyte count (ALC) and absolute monocyte count (AMC) at diagnosis are independent predictors of overall survival (OS) and progression-free survival (PFS) in DLBCL [5]. The combination of both biomarkers into the AMC/ALC prognostic score (AMLPS) stratifies patients into three risk groups: low—(AMC < 630 cells/μL and ALC > 1000 cells/μL), intermediate—(AMC ≥ 630 cells/μL or ALC ≤ 1000 cells/μL) and high-risk (AMC ≥ 630 cells/μL and ALC ≤ 1000 cells/μL) [5]. The AMLPS has been recently validated in several independent studies [6,7] confirming its utility as an assessment tool of prognosis in DLBCL. Post-transplant immunologic reconstitution, particularly ALC recovery (ALC ≥ 500 cells/μL) at day 15, has also been associated with prolonged PFS and OS in multiple hematological malignancies [8–19] and solid tumors [20–22].

However, recent reports suggest that the survival benefit obtained from early lymphocyte recovery post-stem cell transplant in DLBCL patients could be lost with long-term follow-up [10]. Therefore, the aim of this study was to evaluate if day 100 AMLPS (AMLPS-100) affects survival for DLBCL patients in complete remission at day 100 post-APBHSCT. The value of AMLPS-100 was also evaluated as a tool to identify high-risk patients for post-APBHSCT relapse that is simple and could be easily implemented in clinical practice.

## 2. Materials and Methods

### 2.1. Patient Population

DLBCL patients achieving complete remission at day 100 post-APBHSCT at Mayo Clinic, Rochester, MN between 2000 and 2007 were considered for this study. Patients transplanted with bone marrow or combined bone marrow and peripheral blood stem cells and patients with evidence of relapse or progression at day 100 post-APBHSCT were excluded. A total of 134 consecutive DLBCL patients in complete remission at day 100 post-APBHSCT qualified for the study. No patient refused authorization to use their medical records for research and none were lost to follow-up. Approval for the retrospective review of these patients’ records was obtained from the Mayo Clinic Institutional Review Board and the research was conducted in accordance with US federal regulations and the Declaration of Helsinki.

### 2.2. End Points

The primary end point of the study was to assess the impact of AMLPS-100 on OS and PFS by landmark analysis from day 100 in DLBCL patients treated with APBHSCT. The AMC-100, ALC-100, and AMLPS-100 were obtained from a standard day 100 complete blood cell count (CBC). The secondary end point was to evaluate if the AMLPS-100 could stratify DLBCL patients into low-, intermediate- and high-risk groups for OS and PFS post-APBHSCT.

### 2.3. Conditioning Regimen

All patients received carmustine (BCNU) 300 mg/m^2^ on day–6; etoposide 100 mg/m^2^ twice a day on days–5, –4, –3, and –2; cytarabine 100 mg/m^2^ twice a day on days–5, –4, –3, –2; and melphalan 140 mg/m^2^ on day–1 (BEAM).

### 2.4. Prognostic Factors

Prognostic factors evaluated include: age at day 100 (Age-100), ALC-100, AMC-100, absolute neutrophil count at day 100 (ANC-100), gender, International Prognostic Index (IPI) at diagnosis, infused CD34+ cell dose, lactate dehydrogenase at day 100 (LDH-100), hemoglobin at day 100 (Hgb-100), platelets at day 100 (Plts-100), day 15 absolute lymphocyte count post-APBHSCT (ALC-15), and white blood cell count at day 100 (WBC-100).

### 2.5. Response and Survival

Response criteria were based on the guidelines from the International Harmonization Project for Malignant Lymphoma [23]. OS was measured from day 100 to the date of death, or last follow-up. PFS was defined as the time from day 100 to the time of progression, relapse, death, or last follow-up, whichever occurred first.

### 2.6. Statistical Analysis

OS and PFS were analyzed using the approach of Kaplan-Meier [24]. Differences between the survival curves were tested for statistical significance using the 2-tailed log-rank test. The Cox proportional hazard model was used for the univariate and multivariate analysis to evaluate the impact of the variables listed under the prognostic factors section for OS and PFS times [25]. The choice of the cut-off values for ALC-100 and AMC-100 was based on our previous AMLPS publication [5]. *χ*^2^ analysis was used to determine relationships between categorical variables. The Wilcoxon-rank test was used to determine associations between continuous variables and categories, and Spearman correlation coefficients were used to evaluate associations for continuous variables. All two-sided p-values < 0.05 were determined to be statistically significant.

## 3. Results

### 3.1. Patients’ Characteristics

For this cohort of 134 DLBCL patients, the median age at day 100 post-APBHSCT was 57.5 years (range: 23 – 77 years). Sixty-three percent of the patients were males, while 37% were females. The distribution of the patients’ baseline characteristics at day 100 is included in Table 1. The median follow-up period from day 100 post-APBHSCT for the cohort was 5.5 years (range: 0.1 – 12.7 years) and for living patients (N = 93) was 6.9 years (range: 2.5 – 12.7 years). Twenty-seven patients died secondary to relapsed DLBCL. Fourteen patients died of causes not related to DLBCL: 3 patients died of acute respiratory distress syndrome; 3 patients of myelodysplastic syndrome; 2 patients of acute myelogenous leukemia; 2 patients of myocardial infarction; 2 patients of suicide; 1 patient of a motor vehicle accident; and 1 patient of sepsis.

### 3.2. AMC-100, ALC-100, and Survival

**Figure 1.**
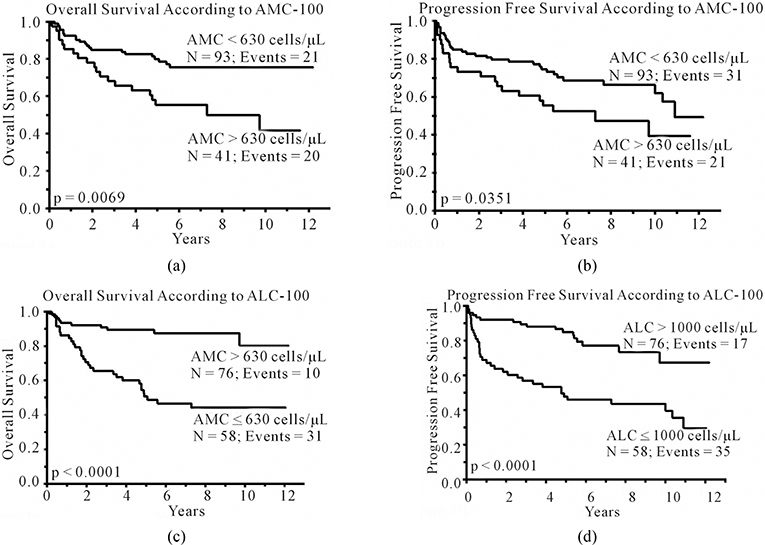
(a) Kaplan-Meier estimates of overall survival from day 100 post-APBHSCT based on day 100 absolute monocyte count (AMC-100); (b) Kaplan-Meier estimates of progression-free survival from day 100 post-APBHSCT based on day 100 absolute monocyte count (AMC-100); (c) Kaplan-Meier estimates of overall survival from day 100 post-APBHSCT based on day 100 absolute lymphocyte count (ALC-100); and (d) Kaplan-Meier estimates of progression-free survival from day 100 post-APBHSCT based on day 100 absolute lymphocyte count (ALC-100).

To determine if AMC-100 and ALC-100 affect survival, we evaluated by univariate analysis both variables as continuous predictors of OS and PFS. As continuous variables both, AMC-100 and ALC-100, predicted OS [Hazard ratio (HR) = 7.16, p < 0.0001 and HR = 0.42, p < 0.009, respectively] and PFS [HR = 5.06, p < 0.0005 and HR = 0.44, p < 0.005, respectively] (Table 2). AMC-100 and ALC-100 were dichotomized using cut-off values previously published [AMC: < 630 cells/μL vs. ≥ 630 cells/μL; ALC: ≤ 1000 cells/μL vs. > 1000 cells/μL] [5]. An elevated AMC-100, defined as ≥630 cells/μL, was associated with inferior OS and PFS on univariate analysis [OS: HR = 2.46, (95% CI, 1.32 – 4.55), p < 0.005; PFS:HR = 1.75, (95% CI, 0.99 – 3.03), p < 0.05]. An ALC-100 ≤ 1000 cells/μL was associated with inferior OS and PFS [OS: HR = 4.85, (95% CI, 2.46 – 10.43), p < 0.0001; PFS: HR = 3.41, (95% CI, 1.93 – 6.25), p < 0.0001]. Figures 1(a) and (b) show superior OS and PFS based on AMC-100 < 630 cells/μL versus AMC-100 ≥ 630 cells/μL [median OS = not reached vs. 7.3 years, 5-year OS rates of 80%, (95% CI 70.4% – 87.1%) vs. 55%, (95% CI, 40.1% – 69.9%), p < 0.007, respectively; and median PFS = 10.9 years vs. 7.3 years, 5-year PFS rates of 75%, (95% CI 64.7% – 82.6%), vs. 55%, (95% CI, 40.1% – 69.9%), p < 0.04, respectively]. Figures 1(c) and (d) show superior OS and PFS based on ALC-100 > 1000 cells/μL versus ALC-100 ≤ 1000 cells/μL [median OS = not reached vs. 5.1 years, 5-year OS rates of 89%, (95% CI, 80.2% – 94.6%), vs. 49%, (95% CI, 36.1% – 61.7%), p < 0.0001, respectively; and median PFS = not reached vs. 4.8 years, 5-year PFS rates of 85%, (95% CI = 74.9% – 91.5%) vs. 46%, (95% CI, 33.6% – 58.9%), p < 0.0001, respectively].

### 3.3. Univariate and Multivariate Analysis

Gender, age-100 (continuous and dichotomized), CD34+, IPI, ALC-15, ALC-100 (continuous and dichotomized), AMC-100 (continuous and dichotomized), and Hgb-100 were identified as predictors for OS and PFS in the univariate analysis (Table 2). In the multivariate analysis, CD34+ and ALC-15 continue to be independent predictors of OS and PFS post-APBHSCT; age dichotomized as < or ≥60 years was associated with PFS. Both AMC-100 ≥ 630 cells/μL and ALC-100 ≤ 1000 cells/μL remained as independent predictors of OS after adjusting for several variables on multivariate analysis, with hazard ratios of 3.83 and 5.46 respectively (p < 0.0002; p < 0.0001); both day 100 variables were independent predictors of PFS (Table 3).

### 3.4. Day 100 AMLPS (AMLPS-100)

**Figure 2.**
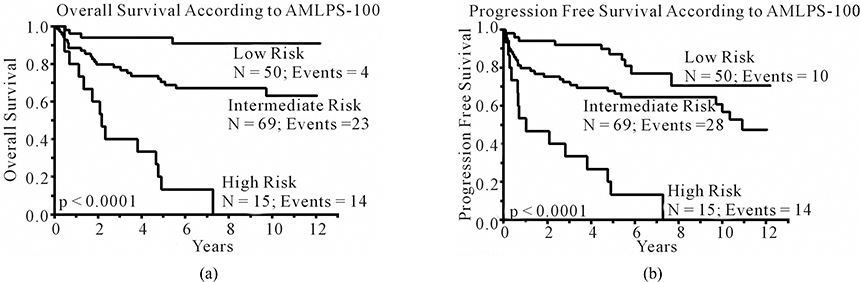
Kaplan-Meier estimates of overall (a) and progression free (b) survival for the entire cohort of patients stratified by the AMC/ALC prognostic score at day 100 (AMLPS-100, stratifying patients into low risk (ALC-100 > 1000 cells/μL and AMC-100 < 630 cells/μL); intermediate-risk (ALC-100 ≤ 1000 cells/μL or AMC-100 ≥ 630 cells/μL); and high-risk (ALC-100 ≤ 1000 cells/μL and AMC-100 ≥ 630 cells/μL).

By univariate and multivariate analysis, the ALC-100 and AMC-100 were independent predictors for OS and PFS post-APBHSCT. Thus, we combinedALC-100 and AMC-100 into day 100 AMC/ALC prognostic score (AMLPS-100), using the same cut-off values from our previous publication of the AMLPS at diagnosis in DLBCL [5], to develop a simple scoring system that can be used to stratify by risk patients with DLBCL that are in complete disease remission at day 100 post-APBHSCT. According to the AMLPS-100, 37.3% (N = 50) of the patients were considered low risk (AMC < 630 cells/μL and ALC > 1000 cells/μL), 51.5% (N = 69) intermediate risk (AMC ≥ 630 cells/μL or ALC ≤ 1000 cells/μL), and 11.2% (N = 15) high risk (AMC ≥ 630 cells/μL and ALC ≤ 1000 cells/μL) (Table 4). Among the groups significant differences were seen in ALC-100, AMC-100, ANC-100, and HgB-100. Patients with a low-risk AMLPS-100 experienced significantly superior OS and PFS compared to the other groups, with a 5-year OS rate of 94% (95% CI, 83.0% – 98.1%); median not reached; p < 0.0001 and a 5-year PFS rate of 87% (95% CI, 74.0% – 94.1%); median not reached; p < 0.0001 (Figures 2(a) and (b)). The estimated 5-year OS among intermediate-risk patients was 70% (95% CI, 58.6% – 80.1%); median not reached (Figure 2(a)) and the 5-year PFS was 68% (95% CI, 56.0% – 77.8%) with a median PFS of 10.9 years (Figure 2(b)). The AMLPS-100 identified a group of high-risk patients with median OS of 2.18 years and an estimated 5-year OS of 13% (95% CI, 3.4% – 40.5%) (Figure 2(a)). Similarly, the median PFS for high-risk patients was 1 year with an estimated 5-year PFS rate of 13% (95% CI, 3.4% – 40.5%) (Figure 2(b)).

## 4. Discussion

Currently, there are no studies available to advise DLBCL patients in complete remission at day 100 post-APBHSCT of their long-term prognosis starting at day 100 post-APBHSCT. We previously published the AMLPS at diagnosis for DLBCL stratifies patients into three risk groups in regard to clinical outcomes. This AMLPS has been validated as an independent prognostic indicator in DLBCL patients by other independent groups [6,7]. Hence, we sought to evaluate if the AMLPS-100 retains its ability to predict clinical outcomes at day 100 post-APBHSCT making it a risk-assessing tool that could be used during follow-up of DLBCL patients in complete remission.

To support the hypothesis that the biomarker AMLPS-100 affects survival in DLBCL patients, it was necessary to demonstrate that both ALC-100 and AMC-100 were associated with clinical outcomes in DLBCL patients in complete remission at day 100 post-APBHSCT. We determined that DLBCL patients presenting with ALC-100 > 1000 cells/μL experienced significantly superior OS and PFS. Similarly, DLBCL patients with an AMC-100 < 630 cells/μL presented superior OS and PFS from day 100 post-APBHSCT.

Since both the ALC-100 and AMC-100 were independent predictors for OS and PFS, we combined them into AMLPS-100. The AMLPS-100 was able to stratify patients into low-, intermediate-, and high-risk groups for OS and PFS post-APBHSCT.

To minimize the inherent biases of a retrospective study, the following steps were taken. With regards to selection bias, we only included DLBCL patients that underwent APBHSCT. Patients infused with peripheral blood as well as bone marrow harvested stem cells were excluded. All patients were treated with the same conditioning regimen. All patients were required to be in complete remission at day 100 for the landmark analysis. With regards to confounding factors, our study included currently known prognostic factors, such as the IPI; in addition, we included Age-100, Hgb-100, ANC-100, LDH-100, WBC-100, and Plts-100, all of which have been reported as prognostic factors at day 100 post-allogeneic stem cell transplantation [1–4].

On the other hand, strengths of this study include the long follow-up period of a well-defined group of DLBCL patients in complete remission at day 100 post-APBHSCT, with a median follow-up from day 100 post-APBHSCT for the cohort of 5.5 years and 6.9 years for living patients. Secondly, AMLPS-100 combines clinical biomarkers for the host immunity (*i.e.*, ALC) [5] and tumor microenvironment (*i.e.*, AMC) [26,27]. Thirdly, the AMLPS-100 is a simple biomarker score obtained from the day 100 CBC post-APBHSCT that can be used to assess clinical outcomes in DLBCL patients in complete remission at day 100 post-APBHSCT, whereas other prognostic techniques such as gene-expression profiling required fresh frozen tissue samples, limiting its use in complete remission DLBCL patients at day 100 post-APBHSCT. Further studies are warranted to validate the AMLPS-100.

## Figures and Tables

**Table 1.** Baseline patients’ characteristics at day 100 (N = 134).

Characteristics	N (%)	Median	Range

Gender			
Male	84 (63)		
Female	50 (37)		
Age-100, years	134 (100)	57.5	(23 – 77)
CD34+	134 (100)	4.43	(2.05 – 14.85)
ALC-15, ×10^9^/L	134 (100)	0.575	(0.02 – 2.4)
AMC-100, ×10^9^/L	134 (100)	0.49	(0.08 – 1.8)
ALC-100, ×10^9^/L	134 (100)	1.045	(0.17 – 5.6)
ANC-100, ×10^9^/L	134 (100)	2.455	(0.22 – 7.8)
WBC-100, ×10^9^/L	134 (100)	4.4	(1.1 – 10.9)
Hgb-100, g/L	134 (100)	11.75	(7.5 – 15.8)
Plts-100, ×10^9^/L	134 (100)	152.5	(17 – 403)
LDH-100, U/L	134 (100)	167.5	(111 – 383)
IPI			
0	13 (9.7)		
1	44 (32.8)		
2	52 (38.8)		
3	21 (15.7)		
4	4 (3)		
AMLPS-100			
Low risk	50 (37.3)		
Intermediate risk	69 (51.5)		
High risk	15 (11.2)		

Abbreviations: ALC = absolute lymphocyte count; AMC = absolute monocyte count; ANC = absolute neutrophil count; WBC = white blood cell count; Hgb = hemoglobin; Plts = platelets; LDH = lactate dehydrogenase; IPI = International Prognostic Index; AMLPS = AMC/ALC Prognostic Score.

**Table 2.** Univariate analysis for overall survival and progression-free survival.

Prognostic factors	Overall survival		Progression-free survival	
	HR (95% CI)	p-value	HR (95% CI)	p-value

Gender		<0.0227		
Male	2.19 (1.11 – 4.70)		1.76 (0.99 – 3.32)	0.0552
Female	0.46 (0.21 – 0.90)		0.57 (0.30 – 1.01)	
Age-100, years (continuous variable)	1.05 (1.02 – 1.08)	<0.0003	1.04 (1.02 – 1.07)	<0.0002
Age at day 100, ≥60 years	2.59 (1.38 – 5.08)	<0.0029	2.54 (1.46 – 4.56)	<0.0009
CD34+	0.84 (0.69 – 0.99)	<0.0404	0.87 (0.74 – 1.01)	0.0631
IPI, ≥2	1.60 (0.85 – 3.13)	0.1508	1.62 (0.92 – 2.93)	0.0940
ALC-15, ×10^9^/L	0.20 (0.07 – 0.52)	<0.0005	0.37 (0.15 – 0.80)	<0.0104
AMC-100, ×10^9^/L (continuous variable)	7.11 (2.86 – 16.08)	<0.0001	5.06 (2.12 – 11.00)	<0.0005
AMC-100 ≥ 630/μL	2.46 (1.32 – 4.55)	<0.0048	1.75 (0.99 – 3.03)	0.0533
ALC-100, ×10^9^/L (continuous variable)	0.42 (0.20 – 0.82)	<0.0094	0.44 (0.22 – 0.79)	<0.0049
ALC-100 ≤ 1000/μL	4.85 (2.46 – 10.43)	<0.0001	3.41 (1.93 – 6.25)	<0.0001
ANC-100 ×10^9^/L	1.13 (0.91 – 1.36)	0.2635	1.11 (0.92 – 1.32)	0.2814
WBC-100, ×10^9^/L	1.05 (0.89 – 1.22)	0.5687	1.04 (0.89 – 1.19)	0.6376
Hgb-100, g/L	0.71 (0.59 – 0.85)	<0.0002	0.78 (0.67 – 0.92)	<0.0026
Plts-100, ×10^9^/L	1.00 (0.994 – 1.001)	0.2590	0.99 (0.99 – 1.00)	0.6292
LDH-100, U/L	1.00 (1.00 – 1.01)	0.2274	1.00 (0.99 – 1.01)	0.3386

Abbreviations: IPI = International Prognostic Index; ALC = absolute lymphocyte count; AMC = absolute monocyte count; ANC = absolute neutrophil count; WBC = white blood cell count; Hgb = hemoglobin; Plts = platelets; LDH = lactate dehydrogenase

**Table 3.** Multivariate analysis for overall survival and progression-free survival.

Prognostic factors	Overall survival		Progression-free survival	
	HR (95% CI)	p-value	HR (95% CI)	p-value

Gender		0.4792		0.4949
Male	1.32 (0.62 – 2.96)		1.24 (0.67 – 2.40)	
Female	0.76 (0.34 – 1.60)		0.80 (0.42 – 1.49)	
Age-100, ≥60 years	1.57 (0.77 – 3.34)	0.2213	2.16 (1.17 – 4.09)	<0.0142
CD34+	0.83 (0.70 – 0.97)	<0.0175	0.89 (0.76 – 1.01)	0.0751
IPI, ≥2	1.88 (0.95 – 3.88)	0.0720	1.90 (1.05 – 3.57)	<0.0341
ALC-15, ×10^9^/L	0.28 (0.09 – 0.074)	<0.0094	0.54 (0.22 – 1.22)	0.1393
Hgb-100, g/L	3.83 (1.89 – 7.85)	<0.0002	2.23 (1.19 – 4.15)	<0.0132
AMC-100, ≥630/μL	3.83 (1.32 – 5.28)	<0.0002	4.30 (2.31 – 8.31)	<0.0001
ALC-100, ≤1000/μL	5.46 (2.99 – 14.10)	<0.0001	5.04 (2.70 – 9.80)	<0.0001

Abbreviations: IPI = International Prognostic Index; ALC = absolute lymphocyte count; AMC = absolute monocyte count; Hgb = hemoglobin.

**Table 4.** Baseline patients’ characteristics based on AMLPS-100.

Characteristics	Low Risk (N = 50)	Intermediate Risk (N = 69)	High Risk (N = 15)	p-value

Gender				
Male	30 (60%)	43 (62%)	11 (73%)	0.6424
Female	20 (40%)	26 (38%)	4 (27%)	
Age-100, years	58.5 (24 – 75)	56(23-76)	62 (41 – 77)	0.2190
CD34+	4.47 (2.05 – 8.23)	4.41 (2.11 – 14.85)	3.82 (2.22 – 9.95)	0.9875
ALC-15, ×10^9^/L	0.68 (0.03 – 2.4)	0.56 (0.02 – 1.89)	0.47 (0.14 – 1.79)	0.2133
AMC-100, ×10^9^/L	0.43 (0.08 – 0.61)	0.50 (0.08 – 1.80)	1.00 (0.63 – 1.65)	<0.0001
ALC-100, ×10^9^/L	1.26 (1.01 – 3.06)	0.91(0.17 – 5.60)	0.71 (0.20 – 0.92)	<0.0001
ANC-100, ×10^9^/L	2.30 (0.27 – 5.25)	2.59(0.28 – 7.80)	3.27 (0.22 – 6.83)	<0.0462
WBC-100, ×10^9^/L	4.4 (1.9 – 9.3)	4 (1.1 – 10.8)	4.6 (1.9 – 10.9)	0.3008
Hemoglobin at day 100, g/L	12.5 (7.9 – 15.8)	11.3 (7.5 – 14.7)	11.8 (8.1 – 13.8)	<0.0027
Platelets at day 100, ×10^9^/L	164 (23 – 373)	139 (17 – 403)	195 (36 – 299)	0.0939
LDH-100, U/L	170 (117 – 286)	166 (111 – 383)	162 (130 – 255)	0.9301
IPI				
0	4 (8%)	9 (13%)	0 (0%)	
1	16 (32%)	26 (36%)	3 (20%)	0.2883
2	23 (46%)	22 (32%)	7 (47%)	
3	6 (12%)	10 (15%)	5 (33%)	
4	1 (2%)	3 (4%)	0 (0%)	

Abbreviations: ALC = absolute lymphocyte count; AMC = absolute monocyte count; ANC = absolute neutrophil count; WBC = white blood cell count; Hgb = hemoglobin; Plts = platelets; LDH = lactate dehydrogenase; IPI = International Prognostic Index.

## References

[1] DeCook LJ, Thoma M, Huneke T, Johnson ND, R. A., Patnaik MM, , “Impact of Lymphocyte and Monocyte Recovery on the Outcomes of Allogeneic Hematopoietic SCT with Fludarabine and Melphalan Conditioning,” Bone Marrow Transplantation, Vol. 48, No. 5, 2013, pp. 708–714. 10.1038/bmt.2012.211 23103674

[2] Bolwell B, Pohlman B, Sobecks R, Andresen S, Brown S, Rybicki L, , “ Prognostic Importance of the Platelet Count 100 Days Post Allogeneic Bone Marrow Transplant,” Bone Marrow Transplantation, Vol. 33, No. 4, 2004, pp. 419–423. 10.1038/sj.bmt.1704330 14688814

[3] Kuzmina Z, Eder S, Bohm A, Pernicka E, Vormittag L, Kahs P, , “Significant Worse Survival of Patients with NIH-Defined Chronic Graft-versus-Host Disease and Thrombocytopenia or Progressive Onset Type: Results of a Prospective Study,” Leukemia, Vol. 26, No. 4, 2012, pp. 746–756. 10.1038/leu.2011.257 21926960

[4] Holtan SG, Hogan WJ, Elliott MA, Ansell SM, Inwards DJ, Porrata LF, , “CD34+ Cell Dose and Establishment of Full Donor Chimerism at Day +100 Are Important Factors for Survival with Reduced-Intensity Conditioning with Fludarabine and Melphalan before Allogeneic Hematopoietic SCT for Hematologic Malignancies,” Bone Marrow Transplantation, Vol. 45, No. 12, 2010, pp. 1699–1703. 10.1038/bmt.2010.49 20208572 PMC7091776

[5] Wilcox RA, Ristow K, Habermann TM, Inwards DJ, Micallef INM, Johnston PB, , “The Absolute Monocyte and Lymphocyte Prognostic Score Predicts Survival and Identifies High-Risk Patients in Diffuse Large-B-Cell Lymphoma,” Leukemia, Vol. 25, No. 9, 2011, pp. 1502–1509. 10.1038/leu.2011.112 21606957

[6] Batty N, Ghonimi E, Feng L, Fayad L, Younes A, Rodriguez MA, , “The Absolute Monocyte and Lymphocyte Prognostic Index for Patients with Diffuse Large-B-Cell Lymphoma Who Receive R-CHOP,” Clinical Lymphoma, Myeloma & Leukemia, Vol. 13, No. 1, 2013, pp. 15–18. 10.1016/j.clml.2012.09.009 PMC4623568 23137719

[7] Keane C, Gill D, Vari F, Cross D, Griffiths L and Gandhi M, “CD4 Tumor Infiltrating Lymphocytes Are Prognostic and Independent of R-IPI in Patients with DLBCL Receiving R-CHOP Chemo-Immunotherapy,” American Journal of Hematology, Vol. 88, No. 4, 2013, pp. 273–276. 10.1002/ajh.23398 23460351

[8] Hiwase DK, Hiwase S, Bailey M, Bollard G and Schwarer AP, “Higher Infused Lymphocyte Dose Predicts Higher Lymphocyte Recovery, Which in Turn, Predicts Superior Overall Survival Following Autologous Hematopoietic Stem Cell Transplantation for Multiple Myeloma,” Biology of Blood and Marrow Transplantation, Vol. 14, No. 7, 2008, pp. 116–124. 10.1016/j.bbmt.2007.08.051 18158968

[9] Porrata LF, Inwards DJ, Ansell SM, Micallef IN, Johnston PB, Gastineau DA, , “Early Lymphocyte Recovery Predicts Superior Survival after Autologous Stem Cell Transplantation in Non-Hodgkin Lymphoma: A Prospective Study,” Biology of Blood and Marrow Transplantation, Vol. 14, No. 7, 2008, pp. 807–816. 10.1016/j.bbmt.2008.04.013 18541201 PMC4494659

[10] Stover DG, Reddy VK, Shyr Y, Savani BN and Reddy N, “Long-Term Impact of Prior Rituximab Therapy and Early Lymphocyte Recovery on Auto-SCT Outcome for Diffuse Large B-Cell Lymphoma,” Bone Marrow Transplantation, Vol. 47, No. 1, 2012, pp. 82–87. 10.1038/bmt.2011.29 21358691 PMC7727309

[11] Porrata LF, Gertz MA, Inwards DJ, Litzow MR, Lacy MQ, Tefferi A, , “Early Lymphocyte Recovery Predicts Superior Survival after Autologous Hematopoietic Stem Cell Transplantation in Multiple Myeloma or Non-Hodgkin Lymphoma,” Blood, Vol. 98, No. 3, 2001, pp. 579–585. 10.1182/blood.V98.3.579 11468153

[12] Boulassel MR, Herr AL, DebEdwards MD, Galal A, Lachance S, Laneuville P, , “Early Lymphocyte Recovery Following Autologous Peripheral Stem Cell Transplantation Is Associated with Better Survival in Younger Patients with Lymphoproliferative Disorders,” Hematology, Vol. 11, No. 3, 2006, pp. 165–170. 10.1080/10245330600667559 17325956

[13] Gordan LN, Sugrue MW, Lynch JW, Williams KD, Khan SA and Moreb JS, “Correlation of Early Lymphocyte Recovery and Progression-Free Survival after Autologous Stem-Cell Transplant in Patients with Hodgkin’s and Non-Hodgkin’s Lymphoma,” Bone Marrow Transplantation, Vol. 31, No. 11, 2003, pp. 1009–1013. 10.1038/sj.bmt.1704050 12774052

[14] Joao C, Porrata LF, Inwards DJ, Ansell SM, Micallef IN, Johnston PB, , “Early Lymphocyte Recovery after Autologous Stem Cell Transplantation Predicts Superior Survival in Mantle-Cell Lymphoma,” Bone Marrow Transplantation, Vol. 37, No. 9, 2006, pp. 865–871. 10.1038/sj.bmt.1705342 16532015

[15] Kim H, Sohn HJ, Kim S, Lee JS, Kim WK and Suh C, “Early Lymphocyte Recovery Predicts Longer Survival after Autologous Peripheral Blood Stem Cell Transplantation in Multiple Myeloma,” Bone Marrow Transplantation, Vol. 37, No. 11, 2006, pp. 1037–1042. 10.1038/sj.bmt.1705373 16708062

[16] Kim H, Sohn HJ, Kim SE, Kang HJ, Park S, Kim S, , “Lymphocyte Recovery Prolonged Survival after Autologous Peripheral Blood Stem Cell Transplantation in T-Cell Non-Hodgkin’s Lymphoma,” Bone Marrow Transplantation, Vol. 34, No. 1, 2004, pp. 43–49. 10.1038/sj.bmt.1704530 15107814

[17] Porrata LF, Gertz MA, Litzow MR, Q Lacy M, Dispenzieri A, Inwards DJ, , “Early Lymphocyte Recovery Predicts Superior Survival after Autologous Hematopoietic Stem Cell Transplantation for Patients with Primary Systemic Amyloidosis,” Clinical Cancer Research, Vol. 11, 2005, pp. 1120–1218. 15709191

[18] Porrata LF, Inwards DJ, Micallef IN, Ansell SM, Geyer SM and Markovic SN, “Early Lymphocyte Recovery Post-Autologous Haematopoietic Stem Cell Transplantation Is Associated with Better Survival in Hodgkin’s Disease,” British Journal of Haematology, Vol. 117, No. 3, 2006, pp. 629–633. 10.1046/j.1365-2141.2002.03478.x 12028034

[19] Porrata LF, Litzow MR, Tefferi A, Letendre L, Kumar S, Gever SM, , “Early Lymphocyte Recovery Is a Predictive Factor for Prolonged Survival after Autologous Hematopoietic Stem Cell Transplantation for Acute Myelogenous Leukemia,” Leukemia, Vol. 16, No. 7, 2002, pp. 1311–1318. 10.1038/sj.leu.2402503 12094255

[20] Ferrandina G, Pierelli L, Perillo A, Rutela S, Ludovisi M, Leone G, , “Lymphocyte Recovery in Advanced Ovarian Cancer Patients after High-Dose Chemotherapy and Peripheral Blood Stem Cell Plus Growth Factor Support: Clinical Implications,” Clinical Cancer Research, Vol. 9, 2003, pp. 195–200. 12538469

[21] Nieto Y, Shpall EJ, McNiece IK, Nawaz S, Beaudet J, Rosinki S, , “Prognostic Analysis Of Early Lymphocyte Recovery in Patients with Advanced Breast Cancer Receiving High-Dose Chemotherapy with an Autologous Hematopoietic Progenitor Cell Transplant,” Clinical Cancer Research, Vol. 10, 2004, pp. 5076–5086. 15297410 10.1158/1078-0432.CCR-04-0117

[22] Porrata LF, Ingle JN, Litzow MR, Geyer S and Markovic SN, “Prolonged Survival Associated with Early Lymphocyte Recovery after Autologous Hematopoietic Stem Cell Transplantation for Patients with Metastatic Breast Cancer,” Bone Marrow Transplantation, Vol. 28, No. 9, 2001, pp. 865–871. 10.1038/sj.bmt.1703236 11781647

[23] Cheson BD, Pfistner B, Juweid ME, Gascoyne RD, Specht L, Horning SJ, , “Revised Response Criteria for Malignant Lymphoma,” Journal of Clinical Oncology, Vol. 25, No. 5, 2007, pp. 579–586. 10.1200/JCO.2006.09.2403 17242396

[24] Kaplan EL and Meier P, “Nonparametric Estimation from Incomplete Observations,” Journal of the American Statistical Association, Vol. 53, No. 282, 1958, pp. 457–481. 10.1080/01621459.1958.10501452

[25] Cox D, “Regression Models and Life Tables,” Journal of the Royal Statistical Society B, Vol. 34, 1972, pp. 187–202.

[26] Diaz-Montero CM, Salem ML, Nishimura MI, Garrett-Mayer E, Cole DJ and Montero AJ, “Increased Circulating Myeloid-Derived Suppressor Cells Correlate with Clinical Cancer Stage, Metastatic Tumor Burden, and Doxorubicin-Cyclophosphamide Chemotherapy,” Cancer Immunology, Immunotherapy: CII, Vol. 58, 2009, pp. 49–59. 18446337 10.1007/s00262-008-0523-4 PMC3401888

[27] Gabrilovich DI and Nagaraj S, “Myeloid-Derived Suppressor Cells as Regulators of the Immune System,” Nature Reviews Immunology, Vol. 9, 2009, pp. 162–174. 10.1038/nri2506 PMC2828349 19197294

